# Conservative Approach in the Management of Large Periapical Cyst-Like Lesions. A Report of Two Cases

**DOI:** 10.3390/medicina57050497

**Published:** 2021-05-14

**Authors:** Roxana M. Talpos-Niculescu, Malina Popa, Laura C. Rusu, Marius O. Pricop, Luminita M. Nica, Serban Talpos-Niculescu

**Affiliations:** 1 Discipline of Restorative Dentistry and Endodontics, Research Center TADERP, Faculty of Dental Medicine, “Victor Babes” University of Medicine and Pharmacy, 300041 Timisoara, Romania; 2 Discipline of Pedodontics, Pediatric Dentistry Research Center, Faculty of Dental Medicine, “Victor Babes” University of Medicine and Pharmacy, 300041 Timisoara, Romania; popa.malina@umft.ro; 3 Discipline of Oral Pathology, Multidisciplinary Center for Research, Evaluation, Diagnosis and Therapies in Oral Medicine, Faculty of Dental Medicine, “Victor Babes” University of Medicine and Pharmacy, 300041 Timisoara, Romania; laura.rusu@umft.ro; 4 Discipline of Oral and Maxillo-Facial Surgery, Faculty of Dental Medicine, “Victor Babes” University of Medicine and Pharmacy, 300062 Timisoara, Romania; pricopmarius@yahoo.com (M.O.P.); talpos@yahoo.com (S.T.-N.)

**Keywords:** periapical cyst, endodontic treatment, calcium hydroxide, decompression, bone regeneration

## Abstract

*Background and Objectives*: Periapical cystic lesions are a pathology frequently addressed to endodontic specialists. Although their therapy is still not standardized, the treatment should be as conservative as possible and by endodontic means, as they are lesions of endodontic origin. The present case report describes two cases of upper central incisors with large cyst-like periapical lesions, and their one-year follow up. *Materials and Methods*: Endodontic orthograde treatment was performed under copious irrigation with sodium hypochlorite, in association with calcium hydroxide as an intra-canal medication for both teeth. Root canal filling was achieved in a separate appointment using the continuous wave of condensation technique. A decompression procedure was used in association with endodontic therapy in the second case to reduce the pressure inside the cystic lesion and to allow its drainage, and only because the root canal could not be dried three weeks after medication. Initial cone beam computed tomography (CBCT) investigations, as well as at the one-year follow up, were used to compare the evolution of the lesion. *Results*: Both cases had a favorable outcome. New bone formation in the periapical region and complete resolution of the lesion was observed at the one-year control in the first case. In the second case, although the lesion was still not completely healed at 12 months, a significant reduction in its size could be observed, showing active signs of healing. *Conclusions*: Endodontic treatment is the first choice option in the management of teeth with pulpal necrosis and large periapical cystic-like lesions. Decompression is the only surgical procedure recommended when the canals cannot be dried and obturated. Large surgical interventions are unnecessary in cases where endodontic treatment can be performed.

## 1. Introduction

Radicular cysts are the most common odontogenic cystic lesions of inflammatory origin involving both the maxillary and the mandibular alveolar bone [1,2,3,4,5]. It is thought that the formation of a radicular cyst is determined by the proliferation and/or degeneration of the epithelial rest cells of Malassez, stimulated by an inflammatory process originating in the pulpal necrosis of a non-vital tooth [5,6]. The majority of apical cysts are asymptomatic and may develop insidiously, being accidently discovered on a routine X-ray control as a large periapical radiolucency involving the apex of one or more teeth [2]. A more precise diagnosis is achieved by histopathological examination, with the confirmation of the lesion as a granuloma or cyst [6,7]. Although conventional radiographic methods cannot be used for a certain diagnosis of periapical cysts, it is considered that round or oval, well-circumscribed radiolucent images of a larger size around the apex of a tooth are cystic lesions [8,9,10]. The use of cone beam computed tomography (CBCT) with superior specificity and excellent precision, can increase the chance of a more accurate preoperative diagnosis [8]; by observing the content of the lesion, especially in apical lesions with a minimum average diameter of 5 mm, CBCT also allows the differentiation between the presence of a semi-solid substance in the lumen or a fluid-containing cavity [8,9,10,11,12].

Two types of peri-radicular cysts have been identified: *true cysts*, with cavities that are completely encircled by an epithelial lining, and with no communication with the apical foramen; and *bay cysts* or *pocket cysts*, with epithelium-lined cavities which connect to the root canals [2,7,13,14], details that might be observed by the clinician on the CBCT examination.

There are no significant clinical, radiological, histopathological, or bacteriological differences between the two types of cysts, except for the morphological relationship with the root canal space. Both types of cysts are associated with intra-radicular root infection (sometimes extra-radicular) [14]. The researchers questioned the need to differentiate them and countered the assumption that the true cyst is a self-entity, and not sustained by the root canal infection [14]; concluding that, regardless of type, the treatment of choice should remain as conservative as possible and using endodontic therapy, and that the elimination of bacteria from the root canal space is of major importance [3,14,15,16,17,18].

Over the years, a cystic lesion may grow in size, remain static, or regress [2]; its growth can involve, not only the periodontal ligament and the alveolar supporting bone, but also adjacent structures, such as the mandibular nerve, the maxillary sinuses, or even the healthy neighboring teeth. In the upper frontal region, large cystic lesions may lead to the involvement of the nasal fossa due to the continuous resorption of the maxillary bone, with implications for the patients breathing and feeding. Moreover, large lesions can produce dental dislocations, pathological fractures, and facial asymmetry [15,16].

Therefore, the early diagnosis and treatment of cystic lesions is of major importance, using either a non-surgical, minimally invasive endodontic orthograde procedure, when possible, or a more complex, combined or surgical approach. The surgical management of cystic lesions implies various procedures, such as decompression [19,20,21,22,23,24,25,26], marsupialization [19,27,28,29], and cystectomy [19,30], and is only indicated in cases where endodontic treatment fails, the canal cannot be dried to complete the three-dimensional obturation of the endodontic system, or endodontic orthograde therapy cannot be performed because of various obstructions present in the root canal [30].

The present paper aims to describe the conservative treatment and the one-year follow up of two cases of large radicular cystic-like lesions of endodontic origin; in the first case, only endodontic therapy in association with calcium hydroxide medication was used, while in the second case, a surgical decompression was necessary in association with the endodontic treatment, to ensure the drainage of the lesion. Both cases showed almost complete healing of the lesions at the one-year follow up.

## 2. Case Reports

**Case 1**

An 18-year-old female patient presented to the dental clinic, with a complaint of a non-painful swelling in the upper front area of the jaw in the last two months. The patient described a history of a single acute episode with pain in the upper anterior teeth that lasted for about 2 days, which had occurred more than 2 years ago. Since then, no provoked or spontaneous pain had occurred, and no signs of swelling had been noticed by the patient until this recent episode.

Intra-oral clinical examination revealed a round to oval swelling, which was located in the anterior right maxillary sulcus, extended onto the buccal labial oral mucosa, and related to both the central and lateral right upper incisors, teeth #11 and #12. The swelling was soft, localized, inflamed, and non-tender to palpation. Significant mesial and distal decay of tooth #11 and discoloration were also observed. Smaller carious lesions and no discoloration were observed on tooth #12.

**Figure 1:**
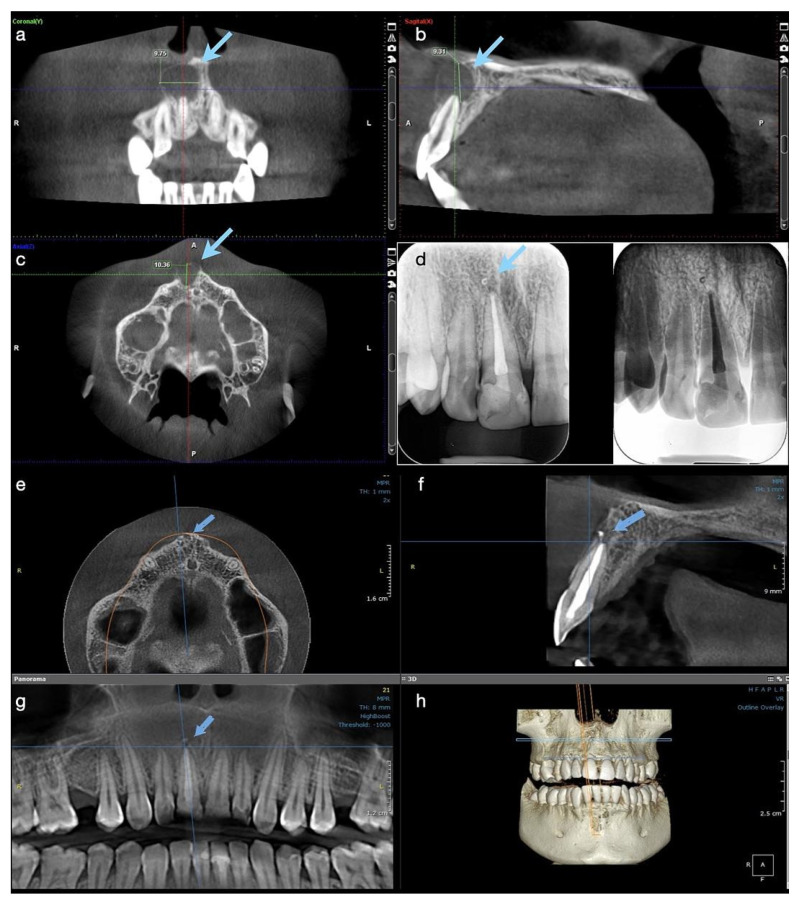
Case 1. Radiological investigations for tooth #11: (**a**–**c**) Initial CBCT scans with the measurements of the lesion’s size, indicated by blue arrows: mesiodistal diameter 9.75 mm, height 9.31 mm, buccolingual diameter 10.36 mm, with the interruption of the cortical buccal plate (9.75 × 9.31 × 10.36 mm); (**d**) control periapical X-ray at 6 months, showing the quality of the endodontic treatment and almost the complete healing of the lesion; (**e**–**g**) CBCT scans at 12 months showing the intact buccal cortical plate, the formation of new bone, and the almost complete healing of the lesion. A small enlargement of the periodontal ligament space is still observed apically, (**h**) 3D reconstruction where intact maxillary bone is observed.

Pulp vitality testing using cold ice showed a negative response only for tooth #11 and a normal response for tooth #12. A negative response was also obtained for tooth #11 for both warm and electric pulp testing. Both teeth presented no tenderness in the long axis percussion test. Periodontal probing was within normal limits for both incisors and the gingiva was intact. The patient had already been subjected to a CBCT investigation with the parameters 11 × 5 cm field of view, 150 µm voxel size, 90 kV, 14 mA, and 5.07 s emission, being a referral case, so the CBCT was analyzed for a precise diagnostic by the endodontist. The images were displayed on a 24” Dell Flat Panel Monitor (Dell, Bucharest, Romania) with 1920 × 1080 pixels resolution. A large unilocular radiolucent lesion, which involved the periapical region of tooth #11, extending distally up to the lateral incisor, without involving its apex, mesially to the middle line of the maxilla, and up to the nasal fossa, with an intact nasal floor, was diagnosed. The size of the lesion was measured using the measurement tools of the Planmeca Romexis software (Planmeca, Helsinki, Finland), and it was about 9 mm in height, 9 mm in the buccolingual direction, and 10 mm in the mesiodistal direction. The lesion appeared well outlined, with a thin radiopaque border on all its contours, but communicated with the apex of the central incisor in its inferior part (Figure 1a–c).

From the history, clinical examination, and radiographic investigation, a diagnosis of pulpal necrosis of tooth #11 complicated with a cystic-like periapical lesion (presumptive pocket cyst) in an acute phase (abscess) was established. Tooth #12 was considered vital with a healthy pulp, responding normally to the cold vitality test, and non-affected by the size of the lesion.

A non-surgical endodontic treatment for tooth #11 was decided on, as being the most conservative procedure, with the continuous monitoring of the lesion at follow-up. The treatment plan was explained to the patient, and her informed consent was taken.

An access cavity was initiated with a high-speed bur along the long axis of the tooth under rubber dam isolation, and immediately a pus discharge was observed. Microsurgical tips were used connected to the suction device of the dental unit to stimulate the drainage. After determination of the working length using hand K-files attached to a Raypex^®^ 6 apex locator (VDW GmbH, Munich, Germany), canals were shaped using a WaveOne^®^ Gold system (Dentsply Sirona, Ballaigues, Switzerland), instrument Medium 35/06, using a crown-down technique with the help of a VDW Silver Reciproc motor (VDW). Abundant irrigation with sodium hypochlorite solution NaClO 5.25% (Chloraxid 5.25%, CERKAMED, Wojciech Pawłowski, Poland) was used from the beginning of the access cavity, alternating with ethylenediaminetetraacetic acid solution EDTA 17% (Endo-Solution 17%, CERKAMED) during the root canal instrumentation. Thirty seconds of ultrasonic activation of the NaClO 5.25% solution was applied at the end with an IrriSafe^TM^ ultrasonic tip K25 (Acteon Satelec, Merignac, France) connected to a piezoelectric ultrasound unit Satelec Suprasson P5 Booster (Acteon Satelec) at a low power setting (3, green) with a vibration frequency of 30 KHz, according to the manufacturer. As the drainage could not be stopped at the end of the treatment and the canal was still wet, calcium hydroxide paste (Calcipast, CERKAMED) was used as an intra-canal medication for the next three weeks.

At the second appointment, the medication was removed, the tooth was re-irrigated and dried, and the obturation was completed with the continuous wave of condensation technique (CWC), using a resin-based sealer (AH Plus Jet^®^, Dentsply Sirona) and Wave One Gold gutta-percha points, and with the help of a EQ-V obturation system (Meta Biomed^®^ Europe GmbH, Mülheim and der Ruhr, Germany). The tooth was adhesively restored with direct composite in the same appointment. No surgical decompression was necessary to lower the pressure and drain the exudate, because the canal was dried in the second appointment and could be obturated.

Post-operative instructions were given to the patient, and she was kept under observation. At the 2-week check-up, a significant reduction in size of the swelling was observed, with no symptomatology of the patient, and after a further month, the swelling had completely disappeared; an increased resistance at palpation in the buccal apical region of the tooth #11 was noticed. After three months, a palpable portion of the buccal bone in the central incisor’s region was observed. At the 6-months follow up, only a control periapical radiograph was taken and a major decrease of the cyst-like lesion, with almost complete healing and formation of new trabecular bone, were observed (Figure 1d).

The patient was recalled at 12 months, and another CBCT with parameters 5 × 5 cm field of view, 85 µm voxel size, 90 kV, 6.3 mA, and 8.70 s emission was performed in the dental clinic to observe the evolution of the lesion. The OnDemand3D (OP 3D™ Pro, KaVo Dental GmbH, Biberach, Germany) program was used for analysis and new measurements on the same computer screen. In all three sections, the lesion appeared almost completely healed; the buccal and alveolar bone were completely restored and the contour of the periodontal ligament was normal, with a small area of enlargement, of only 1.5 mm, around the apex of the tooth (Figure 1e–h). Tooth #12 was still vital, with a normal response. At present, the patient has no symptomatology, the tooth is functional, and the 18-month control will follow.

**Case 2**

A 54-year-old female patient with a non-contributory medical history reported to the dental clinic requesting a full mouth rehabilitation for aesthetic reasons, being unsatisfied with her smile because of the aspect of her frontal teeth. The patient gave a history of an acute, spontaneous painful episode about one month ago, localized in the maxillary frontal area, exacerbated by chewing and touching, and without the possibility of identifying aa specific tooth as the cause.

Intra-oral examination of the upper incisors revealed that tooth #11 was dyschromic and teeth #21 and 22 presented direct composite restorations on the palatal surfaces, possibly as former access cavities for endodontic treatment. Teeth #11, 21, and 22 failed to respond to the thermal and electric vitality testing, while the adjacent teeth responded within normal limits. No pain at percussion was recorded for teeth #21 and 22, while tooth #11 responded slightly positive. Periodontal probing revealed normal limits for all three incisors, and the gingiva was also of normal aspect.

A CBCT investigation was recommended for the upper anterior teeth and performed in the dental clinic with the following parameters: 8 × 15 cm field of view, 250 µm voxel size, 90 kV, 7.1 mA, and 8.14 s emission. On the CBCT images analyzed on the 25” Dell monitor with a 1920 × 1080 pixel resolution, a large unilocular radiolucent lesion which involved the periapical region of tooth #11 was observed, extending towards the mesial region. It exceeded the middle maxillary line and was also involving the mesial part of the apical root third of tooth #21. In an upper direction, it was extending to the nasal fossa, presenting an intact nasal floor.

**Figure 2:**
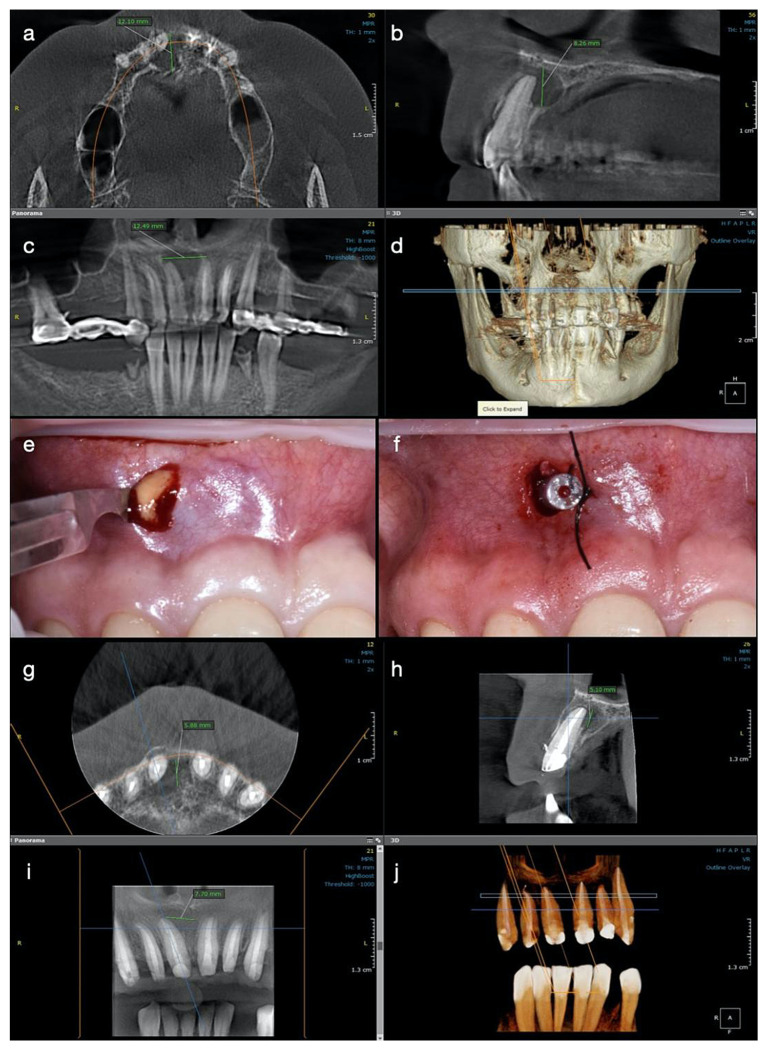
Case 2. Radiological investigations and clinical images of tooth #11: (**a**–**d**) Initial CBCT scans with the measurements of the lesion size: 12.10 mm in buccolingual direction, 8.26 mm in height, and 12.49 mm mesiodistal diameter, with the buccal cortical plate resorbed (12.10 × 8.26 × 12.49 mm); (**e**,**f**) The surgical procedure of decompression at the end of the endodontic treatment with the appearance of yellow fluid draining from the lesion, and the drainage tube fixed with sutures on the mucosa; (**g**–**j**) CBCT scans and the 3D reconstruction at 6 months after the endodontic therapy, with the formation of new bone and increased reduction of the lesion size, starting from its periphery (5.88 × 5.10 × 7.70 mm).

The size of the lesion was measured using the measurement tools of OnDemand3D (OP 3D™ Pro, KaVo Dental GmbH) and was about 15 mm in height, 13 mm in the buccolingual direction, and 13 mm in the mesiodistal. The lesion appeared well contoured by a thin radiopaque border line on all its contours, communicating with the apex of tooth 11 in its inferior part (presumptive diagnosis of pocket cyst) (Figure 2a–d). In addition, on the CBCT scan, teeth #21 and 22 appeared to be endodontically treated, with a radio-opaque obturation material present in the root canal space, 3 mm shorter from the radiographic apex for tooth #21 and 2 mm shorter for tooth #22. A small area of radiolucency of about 1 mm diameter around the apex of tooth #22 was also observed.

From the history, clinical and para-clinical examination, a diagnosis of pulpal necrosis complicated with a cyst-like periapical lesion was established for tooth #11. Teeth #21 and 22 had previously been endodontically treated, with the infra-obturation of the root canal system. Considering the complex prosthetic rehabilitation that followed and the endodontic and periapical status, the decision of an endodontic treatment for tooth #11 was taken, with monitoring of the evolution of the lesion and orthograde endodontic retreatment for teeth #21 and 22. The treatment plan was explained to the patient, and her informed consent was taken.

At the first appointment, tooth #11 was treated. After the access cavity was created under rubber dam isolation using a high-speed round bur, the drainage of a yellow-coloured fluid was observed from the root canal. Suction with microcannulas was used to control the drainage, and working length was determined using hand K-files connected to an apex locator (Raypex^®^ 6, VDW GmbH). The canal was shaped in a reciprocating motion utilizing the instrument Medium 35/06 of the WaveOne^®^ Gold system (Dentsply Sirona), using a crown-down technique. Copious irrigation with sodium hypochlorite solution 5.25% (Chloraxid 5.25%, CERKAMED) was used from the beginning of the access cavity, during the root canal instrumentation in alternation with EDTA solution 17% (EndoSolution, CERKAMED), and several minutes after, to make a total irrigation time of 40 min.

Ultrasonic activation of the irrigant for 30 s was also performed with an IrriSafe^TM^ ultrasonic tip K25 (Acteon Satelec), a Satelec Suprasson P5 Booster (Acteon Satelec) at low power settings (3, green), and a vibration frequency of 30 KHz. At the end of the treatment, the root canal was still wet, so calcium hydroxide paste (Calcipast, CERKAMED) was applied as an intra-canal medication.

At the next appointment, after three weeks, the calcium hydroxide was removed, the tooth was re-irrigated, but continuous secretion in the root canal was present even at the end of the treatment. The decision to replace the medication inside the root canal was taken and the tooth was temporarily restored.

In order to decrease the pressure and to favour the drainage of the exudate from the periapical area of tooth #11, after local anaesthesia, a small incision in the mobile buccal mucosa was performed in the same session with a Bard-Parker surgical blade no. 15, disrupting the cystic wall. Immediately after the incision, a yellow straw-coloured fluid with a dense consistency, containing blood, pus, and cholesterol crystals, was observed draining from the lesion (Figure 2e).

Approximately 10 mL of metronidazole solution 5 mg/mL (Metronidazole B, Braun, Germany) was delivered into the lesion until a clear exudate was observed. A drainage tube was placed into the small window surgically created in the lesion’s buccal wall and was then sutured at its edges, so the drainage remained efficient (Figure 2f). The tube was removed after two days, and two weeks later the patient presented for the completion of the endodontic treatment. After the removal of the intra-canal dressing, the root canal could now be completely dried and obturated using the continuous wave of condensation technique. Post-operative instructions were given to the patient and she was scheduled for recall at three months.

For teeth #21 and 22, endodontic orthograde retreatment was performed in a single visit later on, but no intracanal medication was used. Teeth #12 and 13 were also endodontically treated in one appointment for prosthodontic reasons, after the abutment’s preparation.

At the three-month follow up, the buccal bone in the central incisor’s area became consistent at palpation, and the patient described no symptoms during this period of time or at the clinical exam. At the 6-months control, the patient was subjected to another CBCT investigation. A significant reduction in the size of the periapical lesion in a buccolingual direction, from 12 mm to 5.88 mm, and in a mesiodistal direction, from almost 13 mm to 7.70 mm, was observed with the continued increase in density of the new trabecular bone in all three plans (Figure 2g–j). The total height of the lesion was reduced from 8.26 mm to 5 mm, and the cortical buccal plate appeared significantly wider and mineralized.

**Figure 3:**
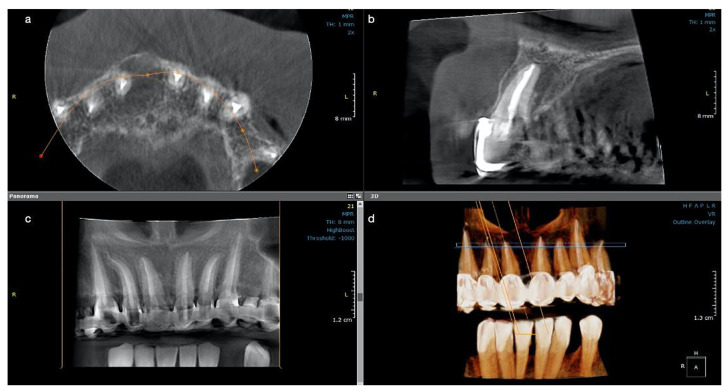
Case 2. CBCT scan for tooth #11 at the one-year control: (**a**) The buccal cortical plate is completely restored, and new bone trabecula can be observed; (**b**) The lesion looks almost completely healed buccally and with a significant reduction in size palatal and height; (**c**) newly formed bone now occupies the cavity of the former lesion; (**d**) the 3D reconstruction shows the quality of the endodontic treatments on all upper frontal teeth.

The postoperative CBCT scan at 12 months revealed the continuous formation of the bone, while the lesion continued to heal, remineralization was observed buccally around the entire root contour, and the palatal radiolucency was also decreased, with the observation of an increased bone regeneration activity and more trabeculae filling the lesion (Figure 3a–d). Both CBCT investigations performed at recall had the same characteristics: 5 × 5 cm field of view, 85 µm voxel size, 90 kV, 6.3 mA and 8.70 s emission, and were analyzed with the same program (OnDemand3D, KaVo Dental GmbH) on the same computer screen as the initial one. At present, the patient is still symptom-free and another clinical and radiological control will follow at 18 months.

## 3. Discussion

Several treatment methods have been described in the management of large periapical cystic lesions of endodontic origin, such as endodontic therapy with or without calcium hydroxide medication, and surgical procedures such as decompression [6,31,32,33,34,35,36,37,38], marsupialization [19,27,28,29], and cystectomy [19,30]; decompression being the least invasive surgical treatment.

Marsupialization, described by Partsch, requires a large window in the cystic wall which is then sutured to the oral mucosa [27,28,29]. Decompression is a more conservative surgical approach, which decreases the intramural pressure, activating the bone deposition within the lesion. Moreover, by exterior drainage of the lesion, it facilitates the complete drying of the root canal, so the endodontic therapy can be finalized with a root canal obturation [20,21,22,23]. As a technique, the decompression can be performed with the use of several tools, such as a drainage tube or stent, which can be placed in a small window created in the lesion’s wall and then sutured to its edges [24]. Cystectomy represents the complete enucleation of the lesion, being a more aggressive and invasive technique and having a high risk of damaging important vital structures [16,27,28]. Moreover, aspiration through the root canal was described as favoring the healing rate of cystic lesions [31]. When selecting the treatment, the clinician should always consider the benefits and risks the procedure can bring to the patient and should choose the minimally invasive one, with the highest success rate and least trauma for the patient [19]. Conservative root canal treatment showed elevated periapical healing rates, suggesting that surgical treatment is unnecessary for a favorable outcome in periapical cysts [13,32,33].

In the present case report, two cases of large periapical cyst-like lesions were successfully treated by using endodontic treatment and calcium hydroxide as intra-canal medication between appointments. The evolution of the lesions was monitored over a period of twelve months with an intermediary control at six months, and showing favorable periapical healing, starting from three to six months.

The advantage of performing endodontic treatment in the healing of periapical lesions is represented by the removal of the infected pulpal tissue and the important reduction of the bacteria inside the root canal. As several antimicrobial agents, such as sodium hypochlorite, are used in endodontics as irrigants to decontaminate and clean the endodontic space, with a broad spectrum and nonspecific killing efficiency against bacteria, spores, and viruses [6,34], endodontic therapy still remains the first alternative in the treatment of periapical cysts. In a surgical procedure without endodontic therapy, the main factors responsible for the development of the lesion remain completely untouched.

In addition, the use of calcium hydroxide as a root canal medication between sessions will increase the disinfection of the endodontic system by neutralizing the remaining microorganisms and will induce a favorable environment for periapical healing [35,36,37]. Moreover, calcium hydroxide [Ca(OH)_2_] is effective against Gram-negative species and inactivates membrane transport mechanisms when performing its antibacterial functions [35,36]. Furthermore, calcium hydroxide has the ability to induce the deposition of hard tissue, favoring the healing of the lesion and bone repair [37].

In both presented cases, calcium hydroxide was used in consideration of these factors and also for controlling the humidity from the root canal, it being known that one of the major problems of orthograde endodontic treatment is the impossibility of drying the root canal in large cystic lesions [32,35,36,37,38,39,40,41].

The period of time required for the root canal medication to be effective varies from two weeks to six months [32,36,40,41]. In the first case, after three weeks of medication, the calcium hydroxide was removed, the canal was dried and obturated, and the tooth restored. No surgical procedure was therefore necessary, although the patient presented a swelling in the first appointment, because the lesion was spontaneously draining as soon as the access cavity was created. Irrigation with sodium hypochlorite during the endodontic treatment in association with aspiration with microcannula inside the root canal increased the removal of the fluids from the periapical area, released the pressure from the lesion, and the tooth could be closed with medication and provisional restoration in the first appointment, and definitively in the second.

Moreover, by using ultrasonic activation of the solution, as in both described cases, the efficiency of the sodium hypochlorite could have been increased [42]. Other means of increasing the efficiency of sodium hypochlorite in removing debris from the root canal or in favoring the healing of large periapical lesions, such as laser activation, have been described in the literature [43,44], but they were not used in the irrigation protocol of the presently described cases.

In the second case, the calcium hydroxide medication was repeated after the first three weeks because the canal was still wet at the end of the procedure with all the supplementary means of irrigation, activation, and suction, and therefore the treatment was completed with a surgical decompression punction in the labial mucosa. The incision favored the drainage of the cystic lesion and also offered the clinician the possibility of observing the cholesterol granules in the exudate, which were a confirmation of its presumptive diagnosis. As the lesion showed active signs of infection with pus draining once the punction was created, metronidazole solution 5 mg/mL, an efficient antibacterial agent with rapid onset of action and efficiency against anaerobic bacteria, was used for irrigation [45,46].

Since histopathology is the only examination that confirms the real nature of periapical lesions, another advantage of using decompression punction is that it allows for a biopsy [9,10,12]. Unfortunately, no histopathological investigation was performed in this case; the assessment of both cases and the diagnosis were established only by clinical examination and precise imagistic radiological methods (CBCT).

In comparison with periapical radiographs, CBCT examination allows more accurate measurements of the lesion limits in all three plans, the observation of its content, and also its direct relation to the root canal lumen [47,48,49]. With these advantages, the clinician is able to establish a presumptive diagnosis closer to the real histopathological one, decide the best treatment plan for the patient, and follow up the evolution of the case.

Moreover, the CBCT examination allows for a better quantification of the lesion size in comparison with periapical radiographs, establishing a more precise periapical index (PAI), the CBCTPAI [47,48]. Moreover, an accurate description of the extension of the lesion into the cortical bone (E) or of the bone destruction (D) can be added to this index [47]. Both described cases showed, according to Estrela et al., a CBCTPAI of 5 + D (lesions bigger than 8 mm, with a destruction D of the buccal cortical bone) [47].

The healing of all periapical lesions with bone destruction starts from the periphery of the defect towards its center, with size reduction caused by the new bone formation, as observed in both present cases. Radiologically, after a period of time, the lesion will appear smaller and bone trabecula will be observed with different radiopacities occupying the space of the former lesion. A complete healing, with a complete restoration of the periodontal ligament architecture on all root contours, can be observed at different periods of time, varying from one to four years [32,50]. In the present case report, at six and twelve months, a significant decrease of the lesion size was observed at the CBCT exam for both treated teeth, showing the favorable outcome of the performed therapy.

A nonsurgical approach should always be adopted for the treatment of endodontic origin cystic lesions, even for the true type of cysts [3,7,14,18]. The decompression and aspiration–irrigation techniques are additionally recommended only for the drainage of the cystic fluid when the humidity inside the root canals cannot be controlled, allowing the endodontist to dry the root canal, and facilitating the three-dimensional filling of the endodontic system. Moreover, their advantage is represented by the minimum discomfort for the patient, being minimally invasive in comparison with large, surgical resective procedures [20,21,22,23].

In both cases, the patients are asymptomatic and still under periodical observation, until the lesions are completely healed.

## 4. Conclusions

Large cystic-like periapical lesions respond favorable to nonsurgical endodontic treatment. Although the evaluation period must be longer when taking into consideration the conservative management of these cases, the major advantages of performing endodontic therapy are represented by the minimally invasive procedures, with high rates of healing for the treated patients. Large surgical interventions are unnecessary in cases where endodontic treatment can be successfully performed.

## Data Availability

Not applicable.
